# Diagnostic Challenges of Tuberculous Lymphadenitis Using Polymerase Chain Reaction Analysis: A Case Study

**DOI:** 10.1155/2015/723726

**Published:** 2015-01-22

**Authors:** Hirokazu Taniguchi, Masahiko Nakamura, Kazuki Shimokawa, Fumi Kamiseki, Shin Ishizawa, Hitoshi Abo, Hideaki Furuse, Takeshi Tsuda, Yasuaki Masaki, Kensuke Suzuki

**Affiliations:** ^1^The Department of Internal Medicine, Toyama Prefectural Central Hospital, Toyama, Toyama 930-8550, Japan; ^2^The Division of Microbiology, Department of Medical Laboratory, Toyama Prefectural Central Hospital, Toyama, Toyama 930-8550, Japan; ^3^The Department of Otolaryngology, Toyama Prefectural Central Hospital, Toyama, Toyama 930-8550, Japan; ^4^The Department of Pathology, Toyama Prefectural Central Hospital, Toyama, Toyama 930-8550, Japan; ^5^The Department of Diagnostic Radiology, Toyama Prefectural Central Hospital, Toyama, Toyama 930-8550, Japan

## Abstract

This report presents a case of tuberculous lymphadenitis that was difficult to diagnose using polymerase chain reaction analysis. An 80-year-old Japanese female was hospitalized due to swollen cervical lymph nodes. Her lymph node tests revealed paradoxical polymerase chain reaction results. Polymerase chain reaction analysis of two biopsy tissues using the Cobas TaqMan revealed a positive result for *Mycobacterium avium* and a negative result for *Mycobacterium tuberculosis*. However, polymerase chain reaction analysis of a cultured colony of acid-fast bacteria from biopsy tissue using the Cobas TaqMan and an alternative polymerase chain reaction analysis of biopsy tissue yielded discordant results. The patient was diagnosed as having tuberculous lymphadenitis. She was treated with antitubercular drugs and subsequently had a reduction in cervical lymph node swelling. Polymerase chain reaction analysis is not 100% accurate; hence, its use as a diagnostic tool for mycobacterial infection requires increased attention.

## 1. Introduction

Tuberculosis is an infection that has affected humankind throughout history. The diagnostic capability for this disease has improved immeasurably during recent years. The definitive diagnosis of mycobacterial infection depends on microscopy, culture, and polymerase chain reaction (PCR) analysis [1–3]. In recent years, PCR analysis has played an important role in Japan because it provides a speedy and exact diagnosis. Cobas TaqMan MTB/MAI is widely used for the detection of the* Mycobacterium tuberculosis* and* Mycobacterium avium* complex, and the technique has high sensitivity and specificity [1, 2]. The Cobas TaqMan MTB/MAI test is a real-time PCR assay for* Mycobacterium tuberculosis* complex,* Mycobacterium avium,* and* Mycobacterium intracellulare* [1].

This case report describes a case of tuberculous lymphadenitis that was difficult to diagnose by PCR analysis.

## 2. Case Report

**Figure 1:**
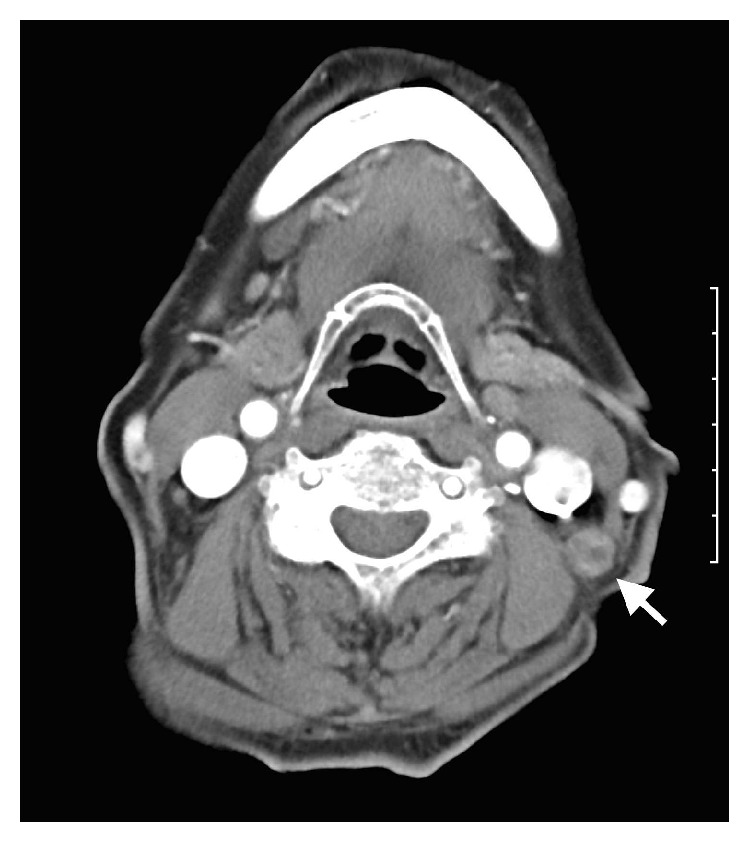
A cervical Computed Tomography scan at the initial examination showed multiple swollen cervical lymph nodes. The arrow indicates one of the swollen lymph nodes.

An 80-year-old Japanese female was hospitalized due to swollen cervical lymph nodes. She had no previous history of TB treatment. Her cervical Computed Tomography scan (Figure 1) findings showed multiple swollen lymph nodes, mainly in the left neck. Furthermore, laboratory studies revealed a serum C-reactive protein level of 0.26 mg/dL and lactate dehydrogenase level of 233 IU/mL (Table 1).

We present an outline of the microbiological findings in Table 2. The patient was suspected of having tumors of the lymph nodes. Three weeks after the first examination, an incision biopsy of a cervical lymph node was performed for diagnostic purposes. The histopathological findings from the biopsy tissue (Sample A) revealed necrotizing granulomas. Therefore, the patient was suspected of having an infection of acid-fast bacteria in the lymph nodes. Laboratory studies revealed a positive enzyme-linked immunospot assay for tuberculosis (ELISPOT) and were negative for immunoglobulin A antibodies against* Mycobacterium avium* complex-specific glycopeptidolipid core antigen (Capilia MAC) (Table 1).

Five weeks after the first examination, an incision rebiopsy of a cervical lymph node (Sample B) was performed for culture and PCR analysis of the tissue. The histopathological findings from the rebiopsy tissue (Sample B) revealed necrotizing granulomas, too. PCR analysis of a biopsy sample using the Cobas TaqMan revealed a positive result for* Mycobacterium avium* and a negative result for* Mycobacterium tuberculosis*. The patient was thus diagnosed as having* Mycobacterium avium* lymphadenitis. Seven weeks after the first examination, clarithromycin 800 mg, rifampicin 450 mg, and ethambutol 500 mg were started for daily administration.

Nine weeks after the first examination, a culture of acid-fast bacteria from rebiopsy tissue (Sample B) was positive in liquid culture medium. A culture of rebiopsy tissue was done at medical laboratory in Toyama Prefectural Central Hospital. PCR analysis of a cultured colony using the Cobas TaqMan revealed a negative result for* Mycobacterium avium* and a positive result for* Mycobacterium tuberculosis*. These findings suggested that the patient not had only* Mycobacterium avium* lymphadenitis but also tuberculous lymphadenitis, and thus isoniazid 300 mg daily was added to her regimen.

Questioning the paradoxical PCR results, we analyzed a remaining frozen specimen from sample A by PCR using the Cobas TaqMan. This analysis revealed a positive result for* Mycobacterium avium* and a negative result for* Mycobacterium tuberculosis*. All PCR analyses were carried out at the same private clinical laboratory testing facility in Japan. Next, an alternative original PCR method on a frozen specimen of sample A was conducted by the Research Institute of Tuberculosis. This PCR analysis of sample A revealed a negative result for* Mycobacterium avium* (negative of IS1311 and DT1) and a positive result for* Mycobacterium tuberculosis* (positive of IS6110). Finally, a separation of viable bacteria in cultured colonies revealed no* Mycobacterium avium* colonies and all* Mycobacterium tuberculosis *colonies by PCR using the Cobas TaqMan. Thus, it was suggested that the patient did not have* Mycobacterium avium* lymphadenitis but tuberculous lymphadenitis, and clarithromycin was discontinued from her regimen.

The patient continued treatment for tuberculous lymphadenitis with antitubercular drugs and experienced a reduction in cervical lymph node swelling.

## 3. Discussion

This case highlights the diagnostic challenges of tuberculous lymphadenitis due to paradoxical results obtained by PCR analysis. PCR analysis of two biopsy tissues using the Cobas TaqMan at a private clinical laboratory testing facility revealed a positive result for* Mycobacterium avium* and a negative result for* Mycobacterium tuberculosis*. However, both a PCR analysis of a cultured colony of acid-fast bacteria from biopsy tissue conducted at a private clinical laboratory testing facility using the Cobas TaqMan and an alternative PCR method of biopsy tissue at the Research Institute of Tuberculosis were negative for* Mycobacterium avium* and positive for* Mycobacterium tuberculosis*.

There are three possible speculations. The first is that the patient had a* Mycobacterium tuberculosis* infection only, and PCR analysis of two biopsy tissues using the Cobas TaqMan revealed a false-positive for* Mycobacterium avium* and a false-negative for* Mycobacterium tuberculosis*. The second is that she had combined infection of* Mycobacterium avium* and* Mycobacterium tuberculosis*, and two parts of biopsy tissues submitted to the clinical laboratory testing facility were infected with* Mycobacterium avium* only, while a part of the biopsy tissue cultured and submitted to the Research Institute of Tuberculosis was infected with* Mycobacterium tuberculosis* only. The third is that she had an infection of* Mycobacterium avium* only, and both PCR analyses of a cultured colony using the Cobas TaqMan and of a biopsy tissue at the Research Institute of Tuberculosis revealed false-negative results for* Mycobacterium avium* and false-positive results for* Mycobacterium tuberculosis*. The second and third speculations should be least likely; therefore, we advocate the first speculation holistically.

A few articles have reported substantial rates of false-positive results by PCR (Cobas Amplicor MTB/MAI) [2, 4, 5]. Bloemberg and coworkers reported that the specificity of the Cobas TaqMan MTB test for nonrespiratory specimens was 94.6% and the accuracy of the Cobas TaqMan MTB test for nonrespiratory specimens might be low, compared with respiratory specimens [2]. PCR analysis is not 100% accurate, and thus its use as a diagnostic tool for mycobacterial infection requires increased attention. Unfortunately, a false-positive result by PCR analysis is difficult to identify. If we receive samples of plural type, we should verify the diagnostic accuracy by conducting PCR analyses in as many of the samples as possible, even if a patient has already received a definitive diagnosis. Furthermore, if we encounter a patient with paradoxical PCR results, we should reexamine the findings using another method without hesitation.

## Figures and Tables

**Table 1:** Laboratory data on initial examination.

Hematology	
White blood cells	6,100/mm^3^
Neutrophils	64.1%
Eosinophils	6.5%
Basophils	0.9%
Lymphocytes	25.4%
Monocytes	3.1%
Red blood cells	347 × 10^4^/mm^3^
Hemoglobin	10.6 g/dL
Hematocrit	33.0%
Platelets	18.1 × 10^4^/mm^3^
Serology	
C-reactive protein	0.26 mg/dL
Biochemistry	
Total protein	6.6 g/dL
Lactate dehydrogenase	233 IU/L
Aspartate aminotransferase	20 IU/L
Alanine aminotransferase	15 IU/L
Alkaline leukocyte phosphatase	209 IU/L
Creatinine	0.8 mg/dL
Enzyme-linked immunospot tuberculosis	Positive
Immunoglobulin A antibodies against *Mycobacterium avium* complex-specific glycopeptidolipid core antigen	Negative

**Table 2:** Outline of microbiological findings.

	Three weeks after first examination	Five weeks after first examination	Nine weeks after first examination	Eleven weeks after first examination	Twelve weeks after first examination	Thirteen weeks after first examination
Sample A	The histopathological findings from an incision biopsy of a cervical lymph node revealed necrotizing granuloma			A PCR analysis of a biopsy tissue revealed a positive for MA and a negative for TB (TaqMan)	A PCR analysis of a biopsy tissue revealed a negative for MA and a positive for TB (RIT)	

Sample B		A PCR analysis of an incision re-biopsy of a cervical lymph node revealed a positive for MA and a negative for TB (TaqMan)	A PCR analysis of a cultured colony revealed a negative for MA and a positive for TB (TaqMan)			A separation of viable bacteria in cultured colonies revealed no MA colonies and all TB colonies

PCR: polymerase chain reaction, MA: *Mycobacterium avium*, TB: *Mycobacterium tuberculosis*, and RIT: The Research Institute of Tuberculosis, Japan Anti-Tuberculosis Association.
